# Bridging Borders, Bridging Barriers: Artificial Intelligence for Dental Education

**DOI:** 10.1111/eje.70141

**Published:** 2026-03-24

**Authors:** Dieter J. Schönwetter, Laura L. MacDonald, Patricia A. Reynolds, Kenneth A. Eaton

**Affiliations:** ^1^ Dr. Gerald Niznick College of Dentistry, Rady Faculty of Health Sciences University of Manitoba Winnipeg Manitoba Canada; ^2^ Dr. Gerald Niznick College of Dentistry and School of Dental Hygiene, Rady Faculty of Health Sciences University of Manitoba Winnipeg Manitoba Canada; ^3^ Faculty of Dentistry, Oral and Craniofacial Sciences King's College London London UK; ^4^ General and Lifelong Learning Division University of Kent Kent UK

**Keywords:** artificial intelligence in education, assistive technology, digital equity, ethical AI implementation, perspectives on AI

## Abstract

The rapid convergence of Artificial Intelligence (AI), Information and Communication Technologies (ICT), and Assistive Technologies (AT) is transforming education, especially its accessibility and inclusivity. This commentary resulted from a think‐tank during a colloquium of academics and practitioners from Europe, Africa, and North America, who came together for two intensive days of keynote presentations, thematic panels, and collaborative workshops to examine AI's promise in healthcare education. Participants highlighted three interwoven themes: employing universal design principles to AI‐driven learning experiences; balancing innovation with digital equity; and crafting policy frameworks that encourage adoption while guarding against algorithmic bias. Yet significant hurdles persist—ethical dilemmas around data privacy, bias in machine learning models, and a digital division between resource‐rich and resource‐poor groups and countries. To address these challenges, the following recommendations emerged: foster interdisciplinary research partnerships, establish transparent AI governance structures, and invest in scalable assistive technologies. Ultimately, continued international collaboration is needed to ensure AI becomes a force for narrowing, not widening, educational disparities—and to secure a future where accessible learning is a reality for all.

## 1 Introduction

In a world increasingly shaped by digital innovation, Artificial Intelligence (AI) is transforming education at all levels. This is evident in health professions education, where AI—alongside Information and Communication Technologies (ICT) and Assistive Technologies (AT)—is revolutionising how knowledge is delivered, assessed, experienced, and created. These tools promise more personalised learning, enhanced clinical simulations, and improved efficiency. Yet the opportunities come with ethical and equity‐related concerns. The rapid adoption of AI risks deepening existing divides through biased algorithms, uneven access, and variable levels of digital literacy. As health education becomes more reliant on AI, critical questions emerge such as: Who gains the most from these advancements and who is left behind? This commentary emerged from a Colloquium on Artificial Intelligence in Health Professions' Education held in 2024 at the University of Brescia.

After this introduction, the commentary presents the background to the use of AI in education. It then highlights gaps in the relevant literature and suggests topics for future research. A description of a think‐tank which took place on the second day of the colloquium follows. The commentary then lists key insights which arose from the colloquium and the implications for dental education^1^ and for future policy. A consideration of conflicts and synergies between perspectives of those who attended the colloquium as well as points of synergy and shared understanding follows. Conclusions and recommendations are then presented.

## 2 Background

Recent scholarship offers a growing body of evidence on the integration of AI in health professions' education, highlighting both enthusiasm for its potential and critical challenges related to equity, ethical use, and implementation.

Awareness and knowledge gaps persist among students and professionals across the medical, dental, and nursing professions, in countries with developed, developing and emerging economies. This indicates a need for improved AI literacy [1, 2, 3, 4, 5]. Positive perceptions and expectations of AI remain high, despite knowledge gaps, with optimism about its potential to enhance diagnostics, education, and research [1, 4, 6, 7]. Demand for educational integration and training is strong, as students widely support the inclusion of AI in curricula and call for structured teaching, resources, and trained faculty [2, 3, 4, 5]. Ethical and regulatory concerns are highlighted, including algorithmic bias, data privacy, and impacts on autonomy, prompting calls for clear ethical guidelines and policies [8, 9]. Generative AI specific impacts are increasingly scrutinised, with studies exploring benefits for learning and writing alongside concerns about misinformation, equity, and implementation disparities [8, 10, 11, 12]. Contextual variations in attitudes, access, and implementation of AI are shaped by local infrastructure and resources, with notable differences in developing and emerging economies [4, 7]. Focus on specific health professions reveals targeted research in dentistry, medicine, nursing, and physical therapy, reflecting discipline‐specific interests in AI's educational potential [3, 7, 8, 13, 14, 15]. Competency frameworks are emerging, with at least one study emphasising the need for clinical AI competencies that blend technical proficiency with ethical and equity‐focused awareness [16]. Barriers to adoption include limited technical infrastructure, inadequate training of personnel, and financial constraints, all of which hinder AI integration in educational settings [4, 15, 17, 18]. Lack of training of personnel remains a major challenge, as the shortage of skilled instructors affects students' preparedness and instructors' willingness to adopt AI tools [4, 15, 18]. High costs of AI software also limit adoption, particularly where access to advanced tools is restricted by financial considerations [4, 7]. Hesitancy or resistance toward AI arises from fears of job displacement, reduced critical thinking, and loss of creativity, contributing to scepticism in many educational and clinical settings [1, 2, 5, 7, 10, 18]. Perceived organisational restrictions further compound resistance, with concerns about institutional policies and professional acceptance limiting AI's educational use [7].

In summary, the literature indicates that while AI is widely viewed as a transformative force in health professionals' education, its adoption is uneven and hindered by structural, ethical, and pedagogical barriers. Literature calls for strategic, inclusive, and context‐aware approaches to integrating AI in ways that support both innovation and equity.

## 3 Gaps in the Literature

AI's integration into the education of health professions promises significant advancements but also presents various challenges. Key gaps in research remain, particularly in understanding AI's long‐term effects, practical implementation strategies, and ethical considerations. Importantly, these gaps exist concurrently with the ever‐increasing “stealth presence” and usage of AI in higher education and in general. The following is a list of these gaps.

### 3.1 Longitudinal Studies on AI Integration

Current research on AI in health education is mostly cross‐sectional, offering limited insight into its long‐term effects. Longitudinal studies are essential to assess AI's impact on student learning, professional skills, and equitable access over time [2]. Reviews in nursing and physical therapy have stressed the need for such studies, as snapshot assessments miss changing perceptions and evolving educational impacts [10, 13]. Without these data, the understanding of AI's long‐term effects remains incomplete, making longitudinal research vital for effective AI policies and pedagogy. Broader higher education reviews call for sustained studies that explore long‐term learner and educator experiences, ethical considerations, and AI integration across disciplines and regions [18]. Without these data, understanding of AI's long‐term effects on accessibility and practice‐readiness remains incomplete. Rigorous, longitudinal research is crucial to shaping effective AI policies, pedagogy, and technology for inclusive education.

### 3.2 Comparative Studies of Faculty and Student Perspectives

Research often focuses on students' experiences with AI but neglects perspectives. A nursing review found only one study which included faculty, highlighting the need for more research on faculty readiness and attitudes toward AI [13]. Similar concerns appear in broader educational reviews, which note the lack of faculty and instructional designer representation in AI research on course design [18]. Understanding both faculty and student experiences is crucial for successful AI adoption, as comparing views can align expectations and address barriers to integration [10, 19].

### 3.3 Practical Strategies and Case Studies for AI Integration

While the importance of AI in health professionals' education is widely acknowledged, there's a clear gap in practical guidance on how to effectively design, implement, and evaluate AI programmes. Studies across disciplines such as medicine, dentistry, nursing, and allied health professions have highlighted the need for curricular reform, given low AI literacy among students and the absence of structured AI education in many programmes [2, 3, 4, 20, 21]. However, these studies often stop at advocacy without offering concrete models or step‐by‐step integration strategies. This gap is further emphasised in reviews that highlight the lack of established frameworks for course design, competencies, and AI integration guidelines. Issues such as inadequate faculty support, limited research on instructor readiness, and a lack of successful implementation examples continue to slow progress [10, 11, 12, 13, 18]. Experts call for collaboration between educators and AI professionals to shift focus from theoretical discussions to actionable frameworks, addressing issues like faculty support and AI integration guidelines [11, 18].

### 3.4 Ethics and AI: Profession‐Specific Guidance Needed

As AI becomes more embedded in healthcare education and practice, there's a growing call for ethical frameworks tailored to specific health professions. Despite widespread discussion of AI ethics, clear, profession‐specific guidelines are still lacking. Two studies stress the urgency of developing regulatory policies, especially for education and training contexts [5, 7]. Ethical education is also key, highlighting the need to prepare healthcare professionals for responsible human‐AI collaboration and to navigate AI's broader social impact [2, 16]. Since ethical challenges vary across fields like dentistry and nursing, frameworks must reflect these differences [1, 15]. There is also the importance of teaching educators how to critically appraise AI‐generated content, alongside developing timely regulations, through inclusive stakeholder engagement [12]. In short, integrating AI ethically into healthcare requires targeted guidance, profession‐specific education, and a strong culture of critical evaluation.

### 3.5 Impact of AI on Lifelong Learning in Healthcare

While AI's potential in formal education is well recognised, its role in lifelong learning and continuing professional development (CPD) for healthcare professionals is still emerging. AI can support personalised learning, deliver real‐time updates, and reinforce ongoing education, yet research in this area remains limited. There is a need for continuing education (CE) courses to keep all professionals, including dental, current with AI trends [3]. There is also a gap in both foundational and advanced AI training [5]. One‐off sessions may not be enough—there is a call for continuous reinforcement to sustain competence [2]. Integration of AI into undergraduate and postgraduate education is essential for all education, but particularly when fostering a culture of lifelong learning [18, 22]. Faculty also need targeted training to effectively teach and apply AI tools [12, 13]. Suggested, current professional development models may need to evolve to build meaningful AI literacy [18]. Innovative solutions—such as the AI framework [7]—offer pathways to personalised, up‐to‐date learning resources. Given the rapid pace of AI change [3, 12], sustained engagement is essential. In short, the future of CPD in healthcare will depend on AI‐integrated education that adapts over time and empowers both learners and educators.

### 3.6 Understanding the Digital Divide in Health Professions Education

The digital divide remains a major barrier to equitable access to AI‐enhanced learning in health professions' education, especially across diverse regions and diverse student populations. Limited access to AI tools, poor internet connectivity, and lack of infrastructure continue to widen educational disparities. Subscription‐based platforms like ChatGPT‐4.0 may deepen this gap, disproportionately affecting students in rural or low‐income areas [11, 18]. Financial constraints and inadequate technical support further limit AI adoption in low‐resource settings, particularly in parts of Africa, where access and research remain minimal [2, 17]. While countries like the U.S. and China dominate AI in healthcare education research, students in developing nations often lack both exposure and digital literacy [11]. Researchers are calling for more studies focused on how digital inequities impact AI‐enhanced learning—especially in under‐represented regions. This includes exploring the lived experiences of students and educators navigating these barriers and ensuring AI‐integrated curricula are inclusive and accessible [12, 17]. Addressing the digital divide is essential for making AI in education accessible and equitable.

### 3.7 Rigorous Evaluation of Generative AI in Education

The rapid integration of AI tools like ChatGPT into health professions' education highlights a pressing need for more robust, empirical research. While interest is growing, most studies to date are qualitative or literature‐based, with a lack of experimental and longitudinal research—particularly in low‐resource settings such as African healthcare education [2, 11, 23]. At the same time, concerns are growing about AI's potential to erode critical thinking and problem‐solving skills essential for practice. Educators stress the importance of designing curricula that balance AI literacy with activities that foster reflection, independence, and deeper learning [12, 18, 19]. To guide responsible integration, there is a clear call for rigorous, outcome‐based studies that track AI's long‐term educational impact. With thoughtful research and design, AI can support—not replace—the development of skilled, thoughtful healthcare professionals.

### 3.8 Ethical Implications

The increasing use of AI tools like ChatGPT in education—especially in health professions—has raised pressing ethical concerns. Key issues include the risk of misuse for cheating, plagiarism, and the erosion of academic integrity. Scholars stress the urgent need for clear policies and ethical frameworks to guide responsible use and prevent harm [5, 12, 19]. Educators and institutions must critically evaluate AI‐generated content and establish safeguards to prevent misuse [11]. Ultimately, timely, well‐defined, and universally accessible guidelines are essential to ensure AI enhances rather than undermines ethical learning environments.

These gaps in AI integration research include the need for longitudinal studies, comparative faculty‐student perspectives, practical strategies, ethical frameworks, and a better understanding of AI's role in lifelong learning and the digital divide. Addressing these gaps will guide more effective, equitable AI adoption in health education.

## 4 Think‐Tank Method: Exploring Perceptions and Experiences of AI in Education

The findings of the think tank, which took place during one session of a two‐day colloquium, are reported in this commentary. Its title was Artificial Intelligence: Opportunities and Challenges. It drew educators, healthcare providers, librarians, students, and policymakers from Europe, Africa, and North America. It is hoped that the commentary will contribute to dialogue and insights about how AI is impacting education. The participants attended knowing it would be focused on AI, from an international perspective, and aimed to disseminate its findings to further discussion on AI. Seventy‐two delegates attended. The colloquium began with a half‐day session with three keynotes on AI in education, the AI World Congress Report, and the perspectives from a Canadian educator. Roundtable discussions on academic integrity, scientific publications and lived experience of AI in the classroom followed. From these discussions, issues emerged for the next day's think tank during which participants joined discussion groups aimed at discerning key themes and challenges (i.e., student‐centred learning and AI; assessment and AI; future considerations using AI transforming learning and teaching with AI, and academic integrity and AI) [24], prioritising, and making recommendations. One to two people in each group documented the discussion for the purpose of generating written records of the think tank. Participants reconvened and shared the discussion outcomes, knowing a team would compose a synthesis culminating in this commentary. In this way, participants explored the transformative potential and inherent risks of AI in health education, drawing from their diverse perspectives. This commentary presents a communal position on the ethical and inclusive integration of AI in health professions education, offering context, insights, and forward‐looking recommendations rooted in equity, universal design, and lifelong learning principles.

## 5 Context and Rationale from the Think‐Tank: The Urgency of AI‐Driven Education

As AI becomes increasingly embedded in academic and clinical education, the need to address issues surrounding AI grows ever more urgent, particularly given disparities and gaps in technological illiteracy, ethics, and access to resources. Hence, participants convened to explore how AI might bridge or exacerbate these gaps. Could AI be harnessed to democratise education and improve global health outcomes? Could it offer greater flexibility and personalisation for learners and educators globally? These were among the central questions that animated the dialogue.

## 6 Key Insights from the Colloquium

The colloquium served as a collaborative space in which participants could explore and critique the integration of AI in health professions education. The following are the themes that emerged from the colloquium, illustrating both the promise of AI and the complexities of its adoption. This process was grounded in a constructivist ontology, which acknowledges the existence of multiple truths shaped by individual experiences and contexts. Epistemologically, it embraces the belief that participants possess valuable, situated knowledge about their own practices. These philosophical underpinnings guided the inquiry, emphasising the importance of co‐constructing knowledge with participants and ensuring that their lived experiences were central to both the discussion feedback presented by the groups and the analysis processes. Moreover, the themes that follow represent an authorial synthesis of diverse viewpoints, aimed at capturing all participants' perspectives.

### 6.1 AI as a Catalyst for Personalised and Person‐Centred Learning

Participants highlighted AI's potential to support person‐centred learning by creating adaptive, responsive learning environments. AI‐powered simulations, personalised feedback systems, and virtual assessments were seen as tools that could improve learner engagement and support clinical skill development. At the same time, attendees emphasised that learners should remain active agents in the learning process, not passive recipients of AI‐curated information. AI can act as a study partner, not a substitute for human interaction or critical thinking.

### 6.2 Efficiency and Innovation in Teaching and Workflow

Educators and clinicians noted that AI technologies could significantly reduce administrative burdens and enhance workflow efficiency. From automating routine grading and administrative tasks to streamlining information searches and improving organisation, AI allows professionals to reallocate time toward higher‐order thinking, mentorship, and innovation in teaching practice. However, there was also a cautionary note: efficiency should not come at the expense of educational depth or the erosion of relational, human elements in teaching.

### 6.3 Ethical Challenges and the Integrity of Learning, and Lifelong Learning

One of the most pressing concerns was academic integrity in an AI world. Participants voiced apprehensions about plagiarism, misuse of AI, and difficulties verifying the authenticity of student work and work in general. Several participants expressed concern that critical thinking skills and intellectual engagement could be undermined if people became over‐reliant on generative AI tools. Ethical dilemmas also extended to data privacy, patient confidentiality, and AI bias—especially in clinical education settings where sensitive data are often used. Calls were made for robust ethical guidelines and institutional and practice policies that address the use of AI in both academic and healthcare environments. Further, it was concluded that academia and the health professions need to reflect collectively on the impact of AI in higher education and practice and how it may be redefining ways of thinking, privacy, ethics, and what constitutes learning.

### 6.4 Digital Equity and Access Barriers

The digital divide between students and institutions with varying levels of technological access and readiness was another theme. AI literacy, digital confidence, and access to reliable internet and devices were identified as equity issues that disproportionately affect underserved or rural populations. Concerns were raised about potential discrimination against those with limited IT skills or access to AI tools—highlighting the need for inclusive design principles and support systems.

### 6.5 Balancing AI Integration With Human Connection

Participants repeatedly returned to the importance of preserving humanity in health education. While AI can accelerate learning and offer new capabilities, it remains limited in replicating emotional intelligence, ethical reasoning, and relational skills central to healthcare practice—at least for now, though it may one day become sophisticated enough to fool some of the people some of the time. Discussions underscored that AI must be used in partnership with humans as a “co‐pilot” not in place of them, and that compassion, mentorship, and reflection should remain core to the educational and healthcare experience.

## 7 Implications for Dental Education

The majority of the participants had dental or general education interests (68 out of 72 attendees). They were aware that integration of AI into dental education has significant implications for academic and clinical training. AI is reshaping how foundational skills and patient‐care competencies are taught and assessed. Dental educators may need to shift from traditional academic lecturing toward effective AI‐enhanced learning experiences to maximise the outcomes of a modern dental curriculum [25, 26].

In preclinical contexts, AI‐enhanced simulation can provide dental students with the opportunity to practice procedures such as cavity preparation in real‐time, with objective feedback that traditional manikins cannot consistently provide. However, AI has been shown to improve dental procedural accuracy and learner confidence, at the learner's own pace. Skill reinforcement is enhanced before students treat patients [27, 28]. In clinical education, AI tools are increasingly being used in diagnostic training such as radiographic interpretation and decision‐making simulations. Such systems support learners' pattern recognition and clinical reasoning without compromising patient safety [28]. However, in the development of dental curricula, there must be a careful balance between AI use to improve manual dexterity, critical thinking, and judgment. AI systems may risk over‐reliance if they are not embedded within robust instructional frameworks [26].

Participants were concerned that AI introduces important considerations for assessment authenticity, academic integrity, and patient safety in dental education. Automated grading systems and adaptive learning analytics can enhance assessment efficiency with scalability and consistent feedback. However, there may be challenges in exercising complex clinical judgement and in ethical reasoning unless augmented with expert dental tutors [28]. As AI becomes integrated into academic curricula, dental schools must adopt policies that uphold academic integrity. They must clarify when and how AI‐generated content is permissible and ensure dental students demonstrate independent clinical reasoning [29]. In using AI tools for improved patient outcomes, students must understand consent, data privacy, and accountability that safeguard patient safety [30]. Dental educators need to learn how AI can best augment, rather than replace, core dental competencies [25, 28]. There was alignment of current literature for AI and dental education with the views held by the think‐tank participants [25, 26, 27, 28, 29, 30].

## 8 Implications for Policy and Future Research

Drawing on the insights generated during the colloquium, several policy implications are proposed to ensure the ethical, inclusive, and future‐focused integration of AI in health professions education and practice. Educational institutions should develop:

- Context‐sensitive AI‐ethics guidelines, addressing areas like data privacy, plagiarism, and student autonomy.
- AI literacy programmes for both educators and students should be prioritised through hands‐on training, workshops, and communities of practice.
- Policymakers and academic leaders must invest in inclusive technology infrastructure to bridge the digital divide, particularly in underserved regions.
- AI tools should align with Universal Design for Learning (UDL) principles to accommodate diverse learning needs and contexts.
- Finally, while AI can support technical and administrative tasks, policies must preserve the humanistic aspects of education, ensuring relational and reflective practices remain central.
  Future research should focus on several key areas to assess the integration of AI in health professions education. First, research must critically evaluate how AI tools influence student learning, engagement, and skill development, especially in diverse international contexts. Studies should also investigate the ethical design of AI, exploring how biases, cultural assumptions, and limitations in training data affect learners from different backgrounds. Longitudinal studies are needed to track the long‐term effects of AI on teaching methods, faculty roles, and student experiences. Given the complexity of AI in education, collaborative, interdisciplinary research teams involving educators, technologists, ethicists, students, and clinicians should be established to design and evaluate AI interventions. Finally, future research should prioritize amplifying people's voices, particularly regarding the psychosocial impacts of AI and their perspectives on its role in teaching and clinical preparation.

## 9 Conflicts and Synergies Between Perspectives

The international and interdisciplinary nature of this colloquium created a dynamic environment where diverse perspectives intersected—sometimes in harmony and at other times in conflict—reflecting the complexity of integrating AI into health professions education and practice in ethical, equitable, and contextually appropriate ways. Optimism and caution emerged as contrasting viewpoints, with some participants embracing AI as a transformative tool for education and practice, while others voiced concerns about its reliability, ethical implications, and potential to depersonalise learning and person‐centred practice. This divide often reflected differing levels of access, training, and comfort with technology. Tensions also arose between efficiency and educational depth, with some participants championing AI's ability to streamline tasks, while others feared that an emphasis on speed and productivity could undermine critical thinking, professional judgement, and empathy. Additionally, the balance between technological advancement and equity was a major point of contention, as participants from under‐resourced regions warned that without careful attention to equity, AI could deepen educational inequalities. Finally, debates about control versus collaboration revealed a central question: who should shape the future of AI in education—governments, institutions, academia, industry, or technology developers? These exchanges highlighted the richness and complexity of integrating AI, emphasising the need for thoughtful, inclusive, and context‐sensitive approaches.

## 10 Points of Synergy and Shared Understanding

Despite the tensions, several areas of strong agreement and shared purpose emerged during the colloquium's discussions. A unanimous commitment to ethical AI use was central, with participants emphasising the importance of establishing clear guidelines, ensuring transparency in AI design, and upholding ethics, integrity and privacy protections. There was also consensus that AI should be viewed as a tool to enhance, not replace, the human aspects of teaching, mentorship, and practice. The growing importance of AI literacy and lifelong learning was recognised across disciplines and continents, with participants agreeing that AI skills must be learned alongside reflective thinking and responsible digital citizenship. Finally, the desire for interdisciplinary and international collaboration was a key point of synergy, with attendees expressing strong enthusiasm for ongoing dialogue and co‐creating solutions that uphold cultural diversity and professional values. These areas of shared understanding underscored a collective commitment to using AI in ways that are ethical, inclusive, and universally relevant.

## 11 Conclusions

The colloquium reaffirmed that AI is not merely a technological tool but a transformative force—one that holds great promise for improving health professions' education, but also one that must be guided by ethical principles, inclusive practices, and human‐centred values. Participants left with a deeper understanding of AI's current capabilities, practical applications, and unresolved challenges. More importantly, they carried forward a shared commitment to building equitable, ethical, and universally relevant strategies for AI integration in health and dental education.

### 11.1 Recommendations—Next Steps for the International Community

To move forward responsibly and inclusively, the international education and health professional practice communities, the following are recommended:

- Fostering ongoing international think‐tanks and communities of practice, both virtually and in person, to sustain dialogue, spark interdisciplinary innovation, and ensure accountability.
- Ensuring policy advocacy and leadership that aligns AI integration with universal design principles, academic freedom, and a commitment to lifelong learning can be supported.
- Amplifying people's voices (students, educators, practitioners, etc.) and integrating perspectives across the world but especially from the global south will be essential in shaping AI policies and educational tools that reflect diverse realities.
- Establishing transparency in AI governance and structure.
- Investing in scalable assistive technology and evaluation to ensure its implementation fidelity and integrity
- Creating and widespread dissemination of open‐access ethical AI frameworks to help guide the use of AI in both academic and practice settings
- Building of inclusive professional development models—with accessible training and mentorship—to empower educators, students, and healthcare providers around the world to engage with AI confidently and competently.

Together, these steps can ensure a more just, inclusive, and innovative future for AI in education and healthcare.

## Funding

The authors have nothing to report.

## Conflicts of Interest

The authors declare no conflicts of interest.

## Data Availability

The data that support the findings of this study are available from the corresponding author upon reasonable request.

## Endnotes

^1^
Dental education is inclusive of all oral healthcare professionals' education.
